# Unveiling the role of CXCL10 in pancreatic cancer progression: A novel prognostic indicator

**DOI:** 10.1515/med-2024-1117

**Published:** 2025-03-21

**Authors:** Xiaochao Wu, Longfei Rong, Ruiyi Tang, Quanpeng Li, Fei Wang, Xueting Deng, Lin Miao

**Affiliations:** Department of Gastroenterology, The Second Affiliated Hospital of Nanjing Medical University, Nanjing, Jiangsu, China; Department of General Surgery, SIR RUN RUN Hospital of Nanjing Medical University, Nanjing, Jiangsu, China

**Keywords:** pancreatic cancer, weighted gene co-expression network analysis, hub genes, CXCL10, VEGFA, macrophages

## Abstract

**Objective:**

Pancreatic cancer is distinguished by its high likelihood of metastasis and drug resistance, while the fundamental mechanisms are inadequately elucidated. This study aimed to identify pivotal hub genes associated with pancreatic cancer and assess their potential utility in predicting its onset and progression.

**Methods:**

Weighted gene co-expression network analysis (WGCNA) combined with differential expression analysis identified novel susceptibility modules and hub genes for pancreatic cancer. Kyoto Encyclopedia of Genes and Genomes and gene ontology analyses were utilized to explore the potential roles of these hub genes. Receiver operator characteristic curves and nomogram models were developed to evaluate diagnostic efficacy. Mendelian randomization, flow cytometry, Transwell, and RNA sequencing were conducted to explore the association between C-X-C motif chemokine ligand 10 (CXCL10) and immune infiltration.

**Results:**

WGCNA analysis was performed to build gene co-expression networks, and ten key genes were found. CXCL10 was the central gene, and its expression was significantly linked to the survival of patients with pancreatic cancer and their response to immune checkpoint inhibitors. CXCL10 demonstrated the ability to stimulate the differentiation of macrophages toward the M2 phenotype. CXCL10 could facilitate the metastasis of pancreatic cancer cells by modulating macrophage polarization. CXCL10 affects macrophage polarization by regulating the expression of vascular endothelial growth factor A.

**Conclusions:**

CXCL10 demonstrates potential as a therapeutic target for managing pancreatic cancer.

## 1 Introduction

Pancreatic cancer, characterized by its aggressive nature and rapid progression, poses significant challenges in early detection and treatment, resulting in a low 5-year survival rate [1,2]. The disease’s elusive characteristics, lack of specific biomarkers, and swift advancement often lead to missed opportunities for timely diagnosis and intervention [3,4]. Most individuals are beyond the stage for potentially curative surgery at the time of diagnosis, leading to dismal survival rates. Therefore, identifying specific biomarkers and a deeper understanding of the molecular underpinnings of pancreatic cancer hold immense significance for its clinical diagnosis and management.

Identifying distinct gene expressions in pancreatic cancer is crucial in elucidating its complex mechanisms, detecting diagnostic indicators, and evaluating treatment and prognosis [5]. Bioinformatics tools such as weighted gene co-expression network analysis (WGCNA), Kyoto Encyclopedia of Genes and Genomes (KEGG) enrichment analysis [6], and gene set enrichment analysis significantly aid in discovering disease-related pathways. WGCNA is widely employed in proteomics, genetic markers, gene expression profiles, metabolomics, and other complex datasets to help identify potential therapeutic targets and biomarker candidates [7].

Advancements in tumor research, particularly in tumor immunology, highlighted the significant impact of the tumor immune microenvironment on tumor progression [8,9]. The tumor immune microenvironment primarily comprises immune cells, cytokines, and other components essential in tumor growth, progression, and metastasis. Immune cells, such as T cells and macrophages, modulate the proliferation and metastasis of tumor cells through secreting cytokines and other mechanisms, influencing tumor progression [10–12]. Tumor-associated macrophages (TAMs) are the most prevalent immune cells in tumors [13]. As key players in the innate immune system, macrophages perform phagocytosis, pathogen elimination, and immune signaling. Macrophages are crucial for inflammation defense, tissue development, and homeostasis. Under the influence of the complex tumor microenvironment, macrophages can be recruited to the tumor area to be polarized into the M1 state, inhibiting tumor growth or the M2 state and promoting tumor growth [14–16]. TAMs can polarize into M2 under the influence of hypoxia, cytokines in the tumor microenvironment, or extracellular vesicles [17]. The relative increase in the M2 TAMs is closely related to poor 5-year survival rates in cancer patients [18]. Exploring various factors affecting the M2 polarization status of macrophages in the tumor immune microenvironment is crucial.

This study employed WGCNA to construct a co-expression network, identifying cancer-associated central genes. The core gene C-X-C motif chemokine ligand 10 (CXCL10) exhibited a close association with the development of pancreatic cancer. Reportedly, elevated CXCL10 expression correlates with poor prognosis in pancreatic cancer; however, the mechanisms remain unclear. Our findings indicate that CXCL10 potentially facilitates the progression of pancreatic cancer by modulating the expression of vascular endothelial growth factor A (VEGFA), thereby inducing macrophage polarization towards the M2 phenotype. Our research offers compelling evidence supporting the potential of CXCL10 as a viable target for clinical diagnosis and treatment of pancreatic cancer.

## 2 Materials and methods

### 2.1 Data

We gathered clinical features and genetic expression information for pancreatic cancer specimens from the primary source, the Gene Expression Omnibus (GEO) database, by extracting microarray data on mRNA expression from series GSE55643.

### 2.2 Identification of differentially expressed genes (DEGs)

We loaded, corrected, and normalized the dataset GSE55643 using R software (version 4.3.1). The method was consistent with the previous study [9]. DEGs were screened using the “Limma” package. Following the analysis of expression level significance, “pheatmap” and “ggplot2” packages in R were used to create volcano plots and heatmaps of DEG expression.

### 2.3 Analysis of gene co-expression networks with weights

WGCNA is a systematic approach commonly used to identify genetic relationships among different samples and pinpoint genomes that collaborate closely. Leveraging the interconnection of genomes and their correlation with phenotypic traits, WGCNA facilitates pinpointing potential marker candidates [19]. We used the “WGCNA” software in R to construct a gene co-expression network tailored to pancreatic cancer. This study assessed the link between distinct modules and the disease mechanism of pancreatic cancer, focusing on the module that emerged as the pivotal gene set from WGCNA analysis.

### 2.4 Screening key candidate genes and gene ontology (GO)/KEGG analyses

Genes located at the intersection were considered possible hub genes involved in the progression of pancreatic cancer. KEGG is a detailed database for the organized analysis of gene functions [20]. GO analysis and KEGG pathway enrichment were performed using the “clusterProfiler” tool in R to understand the mechanisms behind disease progression and pathogenesis.

### 2.5 Protein–protein interaction (PPI)

The STRING database and Cytoscape software platform were used to predict and visualize molecular interactions and PPI networks. In Cytoscape, the degree algorithm was used to prioritize significant genes within the PPI networks.

### 2.6 Nomogram

A nomogram model for evaluating pancreatic cancer risk was developed using the “rms” package, following the methodologies described by Park [21]. The model’s accuracy was assessed using Harrell’s concordance index to determine its predictive capability. The accuracy of the suggested biomarkers was confirmed by creating a receiver operator characteristic (ROC) curve using the “ROC” software. The model’s accuracy was measured using the area under the ROC curve (AUC), where an AUC value between 0.9 and 1 indicated exceptional precision.

### 2.7 Immune cell analysis

CIBERSORT was used to assess the extent of immune cell infiltration in 22 immunocytes from the pancreatic cancer cohort [22].

### 2.8 Clinical prognostic analysis

RNA-sequencing expression (level 3) profiles and corresponding clinical information for pancreatic cancer were downloaded from the Cancer Genome Atlas Program (TCGA) dataset (https://portal.gdc.com). Univariate and multivariate Cox regression analyses were performed to identify the proper terms to build the nomogram. The forest was used to display the *p*-value, hazard ratio (HR), and 95% confidence interval (CI) of each variable through the “forestplot” R package. A nomogram was developed based on the multivariate Cox proportional hazards analysis to predict the X-year overall recurrence. The nomogram provided a graphical representation of the factors that could be used to calculate the risk of recurrence for an individual patient by the points associated with each risk factor through the “rms” R package.

### 2.9 Tumor immune dysfunction and exclusion (TIDE)

RNA-sequencing expression (level 3) profiles and corresponding clinical information for pancreatic cancer were downloaded from the TCGA dataset (https://portal.gdc.com). Patients were divided into high expression of CXCL10 (G1 = 90) and low expression of CXCL10 (G2 = 89). Immune checkpoint blockade therapy (ICB) response was assessed using the TIDE algorithm. TIDE evaluates two tumor immune escape mechanisms: dysfunction of cytotoxic T lymphocytes and rejection. Potential ICB response was predicted using the TIDE algorithm. A high TIDE score means poor efficacy of ICB, and a short survival time after treatment [23].

### 2.10 Mendelian randomization (MR)

Data were obtained from an open database. Two samples were compared to explore the potential cause-and-effect relationship between a central gene and the risk of pancreatic cancer, utilizing single nucleotide polymorphisms (SNPs) as instrumental variables. In MR analysis, the key gene CXCL10 and pancreatic cancer were selected from the genome-wide association studies (GWAS) data that was accessible to the public. Information regarding CXCL10 is available at https//gwas.mrcieu.ac.uk/datasets/prot-c-4141_79_1/. Data on pancreatic cancer can be accessed at https//gwas.mrcieu.ac.uk/datasets/ebi-a-GCST90018893/. Two-sample MR analysis was performed using “TwoSampleMR.”

### 2.11 Cell culture

THP-1 cells were acquired from Abbkine (Abbine Inc., China). PANC-1 cells were sourced from Pricella (Procell Life Sciences & Technology Co., Ltd, China). THP-1 cells were cultured in 1640 medium treated with 1% penicillin-streptomycin (Biosharp, China) and 10% fetal bovine serum (FBS; ExCell, Uruguay). PANC-1 underwent culturing in Dulbecco’s modified Eagle’s medium (Gibco, USA) treated with 1% penicillin-streptomycin (Biosharp, USA) and 10% FBS (ExCell, Uruguay).

### 2.12 Polarization of macrophages

THP-1 cells were used for planking along with Phorbol 12-myristate 13-acetate (Multi Sciences, 100 ng/mL) to induce differentiation into mature macrophages. Following 48 h of cultivation, the cells exhibited complete adhesion to the substrate, transitioning from a suspended state to an adherent phenotype. Next, THP-1 cells were treated with CXCL10 (MedChemExpress, 100 ng/mL) for 4 h. The cells were rinsed and cultured with a new medium for 1 day. The resulting media was gathered for flow cytometry analysis.

### 2.13 Flow cytometry assay

Collected THP-1 cells were incubated with Fixable Viability Kit (BioLegend, USA), Pacific Orange anti-human BV510 antibody (BD Pharmingen, USA), PerCP/Cy5.5 anti-human CD45 antibody (eBioscience, USA), PE/Cyanine7 anti-human F4/80 antibody (BioLegend, USA), FITC anti-human CD11b antibody (eBioscience, USA), and APC anti-human CD86 antibody (BD Pharmingen, USA). After fixation and permeabilization of cells using the Fixation/Permeabilization Solution Kit (Biolegend, USA), PE anti-human CD206 antibody (BD Pharmingen, USA) was applied to stain intracellular markers.

### 2.14 Transwell migration assay

The cell migration examinations employed Transwell inserts with an 8 µm pore size (Corning, USA) positioned in 24-well plates. PANC-1 cells were placed in the top chambers with 400 µL of new media without FBS in co-culture experiments. The lower chambers were loaded with 600 µL of THP-1 cells (control and CXCL10-treated) suspension with 10% FBS. After incubating for 48 h, non-migratory cells were located in the upper chamber. The migrated cells on the lower surface of the chamber were fixed with methanol for 20 min and stained with 0.1% crystal violet for 30 min. The cells were rinsed with water and quantified in 4–5 randomly selected areas using a microscope.

### 2.15 Transfection

PANC-1 cells were seeded in 24-well plates at a density of approximately 1:36 per well when the confluence of cells in 10 cm culture dishes reached 70–80%. Once the confluency of the plates approached 60%, small interfering RNAs (siRNAs) were transfected into the cells using Lipofectamine 3000 (Invitrogen, USA) following the manufacturer’s instructions.

### 2.16 RNA extraction and real-time quantitative PCR (qPCR) analysis

Total RNA from PANC-1 was isolated by TRIzol reagent (Vazyme, China) according to the manufacturer’s protocol. Complementary DNA (cDNA) was synthesized using a reverse transcription kit (Vazyme, China). The expression levels of CXCL10 were quantified utilizing a qPCR kit (Vazyme, China). The resultant data were exported in Excel format. The relative expression levels of each gene were calculated and analyzed using the 2^–ΔΔCt^ method. Statistical analyses were conducted using GraphPad software. All mentioned sequences are listed in Table S1.

### 2.17 RNA sequencing and analysis

Total RNA was extracted using TRIzol reagent (Thermo Fisher, USA) according to the manufacturer’s procedure. The total RNA quantity and purity were analyzed using Bioanalyzer 2100 and RNA 6000 Nano LabChip Kit (Agilent, USA). The sequencing library was constructed with high-quality RNA samples with RIN number >7.0. After extracting total RNA, mRNA was purified from total RNA (5 µg) using Dynabeads Oligo (dT) (Thermo Fisher, USA) with two rounds of purification. Next, the mRNA was split into short fragments using divalent cations under elevated temperature; magnesium RNA Fragmentation Module (NEB, USA) under 94℃ for 5–7 min. The cleaved RNA fragments were reverse-transcribed to create the cDNA using SuperScript™ II Reverse Transcriptase (Invitrogen, USA). It was then used to synthesize U-labeled second-stranded DNAs with *E. coli* DNA polymerase I (NEB, USA), RNase H (NEB, USA), and dUTP solution (Thermo Fisher, USA). An A-base was added to the blunt ends of each strand, preparing them for ligation to the indexed adapters. Each adapter contained a T-base overhang for ligating the adapter to the A-tailed fragmented DNA. Dual-index adapters were ligated to the fragments, and size selection was performed using AMPureXP beads. The U-labeled second-stranded DNAs were treated with heat-labile UDG enzyme (NEB, USA). The ligated products were amplified by PCR under the following conditions: initial denaturation at 95°C for 3 min; eight cycles of denaturation at 98°C for 15 s, annealing at 60°C for 15 s, extension at 72°C for 30 s, and final extension at 72°C for 5 min. The average insert size for the final cDNA libraries was 300 ± 50 bp. Finally, 2 × 150 bp paired-end sequencing (PE150) was performed using an Illumina Novaseq™ 6000 (LC-Bio Technology CO., Ltd, Hangzhou, China) following the vendor’s recommended protocol.


**Ethical approval:** TCGA and GEO databases are public databases. All patients involved in the databases have received ethical approval, and relevant data may be downloaded for research purposes for free. All procedures were performed in accordance with the relevant guidelines and regulations since this is not a clinical trial. Therefore, ethics approval and consent for participation are inapplicable.

## 3 Results

### 3.1 DEG screening

**Figure 1:**
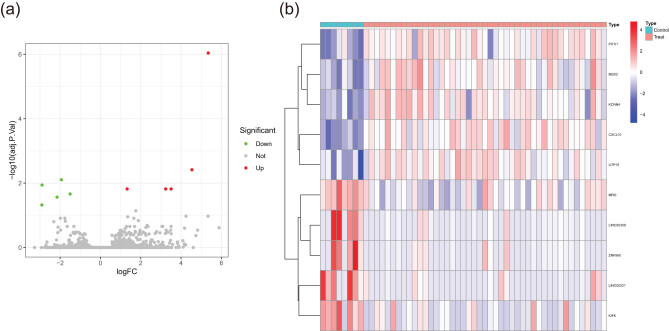
DEGs screening. (a) Volcanic map for differential expression analysis of GSE55643. (b) Heatmap for differential expression analysis of GSE55643. Blue represents downregulated genes, red represents upregulated genes, and black represents undifferentiated genes.

The pancreatic cancer dataset (GSE55643) was obtained from the GEO database. DEGs in tumors and adjacent tumors were identified, and ten DEGs were identified in the tumors compared to adjacent tumors, five upregulated and five downregulated genes (Figure 1a and b and Table S2).

### 3.2 Construction of the WGCNA network and identification of pancreatic cancer-related module

**Figure 2:**
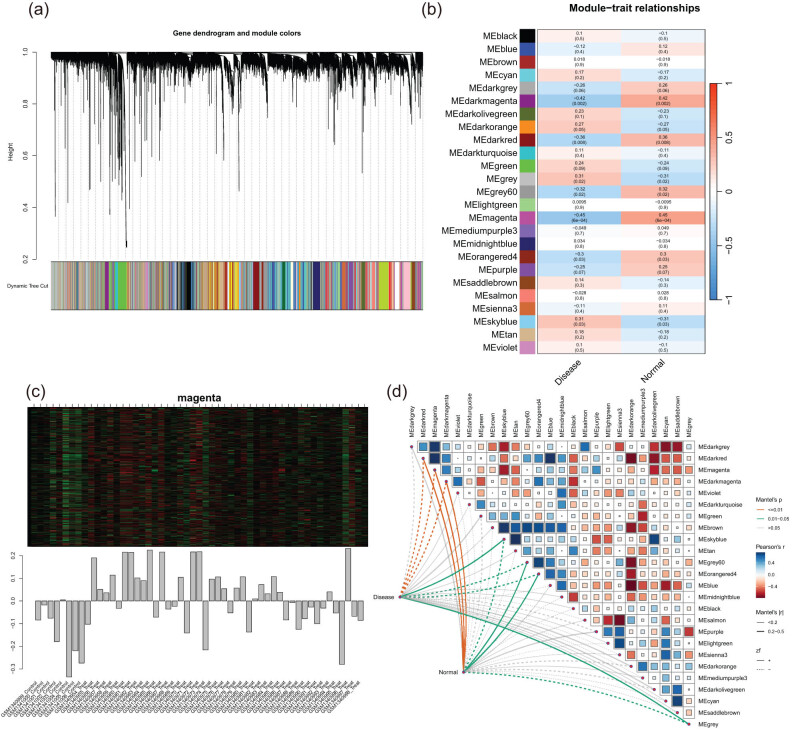
Construction of WGCNA network and identification of pancreatic cancer-related module. (a) Dendrogram of all genes in the GSE55643 dataset was clustered based on a topological overlap matrix (1-TOM). Each branch in the clustering tree represents a gene, while co-expression modules were constructed in different colors. (b) Module-trait heatmap of the correlation between the clustering gene module and pancreatic cancer in the GSE55643 dataset. Each module contains the corresponding correlation coefficient and *p*-value. (c) Sample distribution in the magenta module. (d) Modules corresponding to pancreatic cancer tissue and normal tissue.

To investigate the correlation between potential gene modules and pancreatic cancer, WGCNA was performed on the candidate genes in the pancreatic cancer-specific dataset (GSE55643) (Figure 2a). We identified 25 distinct modules (Figure 2b, *p* = 6 × 10^−4^), and the magenta module exhibited the most significant difference (Figure 2c). After evaluating positive correlation coefficients, the magenta module was chosen from the GSE55643 dataset (Figure 2d and Table S3).

### 3.3 Function of hub genes

**Figure 3:**
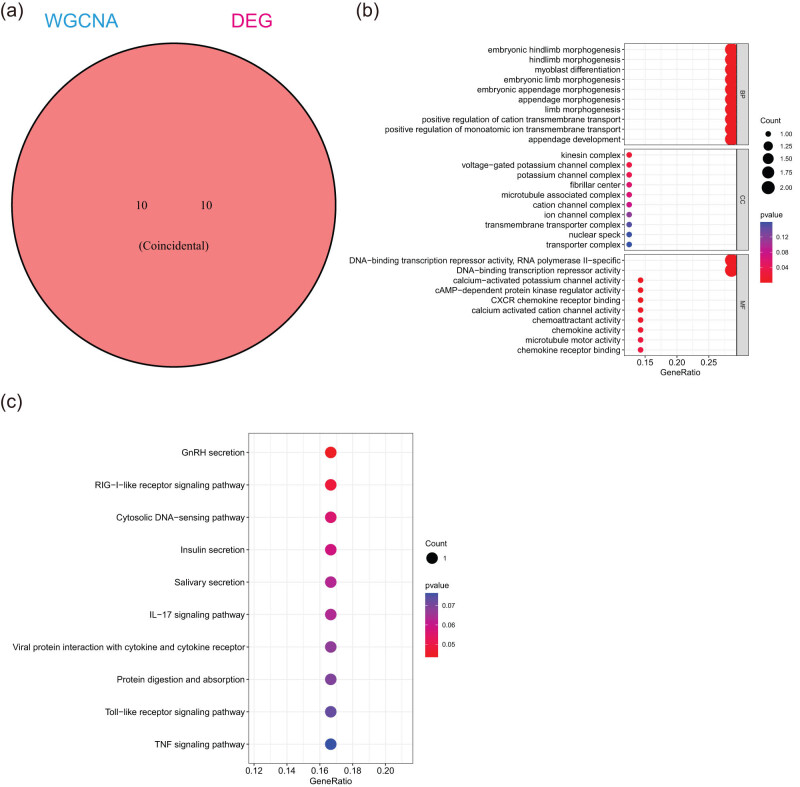
Function of hub genes. (a) The Venn diagram revealed 10 overlapping candidate hub genes. (b) GO enrichment analysis of candidate hub genes. (c) KEGG pathway analysis of candidate hub genes.

We identified 10 overlapping genes as potential hub genes that may be involved in the progression and development of pancreatic cancer by examining co-expressed genes between WGCNA-derived hub genes and DEGs (Figure 3a). The DEGs were enriched in DNA-binding transcription repressor activity, CXCR chemokine receptor binding, and chemokine activity (Figure 3b). The KEGG analysis of DEGs revealed variations in the secretion of GnRH, the signaling pathways of RIG-I-like receptors, cytosolic DNA-sensing, and interleukin-17 (Figure 3c). Signaling pathways are closely related to the occurrence and development of various tumors [24–27].

### 3.4 A nomogram model for predicting pancreatic cancer risk

**Figure 4:**
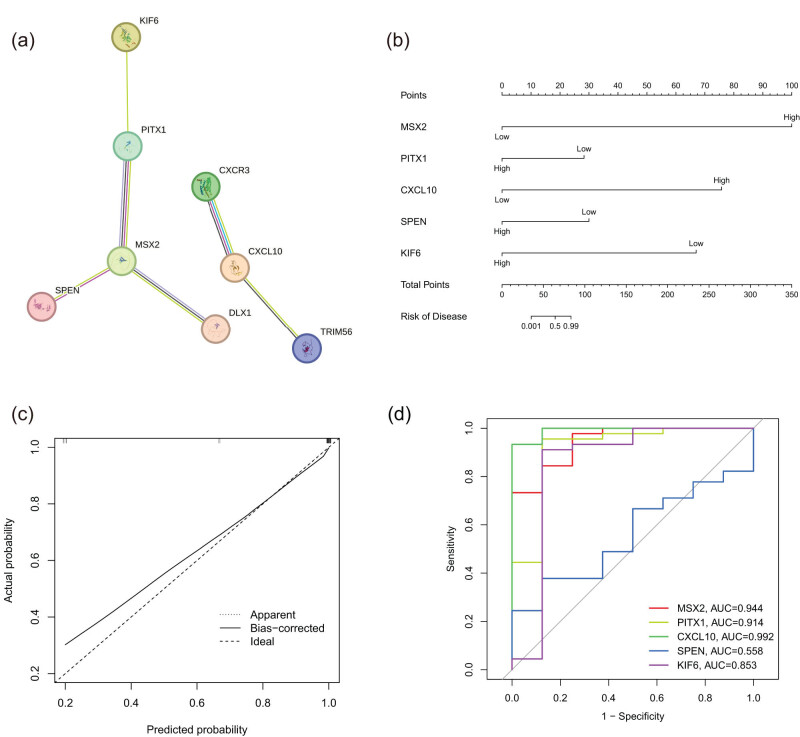
A nomogram model for predicting pancreatic cancer risk. (a) PPI network of overlapping hub genes. (b) Nomogram model of hub genes. (c) Calibration of hub genes. (d) ROC curves to assess the diagnostic efficacy of the nomogram model and each hub gene.

We further evaluated the role of these key genes in the prognosis of pancreatic cancer. STRING, an online tool, was used to build the intersecting hub gene PPI network (Figure 4a). A nomogram was developed to estimate the risk of pancreatic cancer (Figure 4b). Our model demonstrated high accuracy in predicting pancreatic cancer. The diagnostic capability of five central genes (MSX2, PITX1, CXCL10, SPEN, and KIF6) was analyzed through ROC and a calibration curve highlighting the exceptional predictive accuracy of these genes (Figure 4c). The nomogram’s AUC effectively distinguished pancreatic cancer cases and controls (Figure 4d). The AUC values for MSX2, PITX1, CXCL10, SPEN, and KIF6 were notably 0.944, 0.914, 0.992, 0.558, and 0.853, respectively.

### 3.5 CXCL10 could be a standalone predictor for pancreatic cancer prognosis

**Figure 5:**
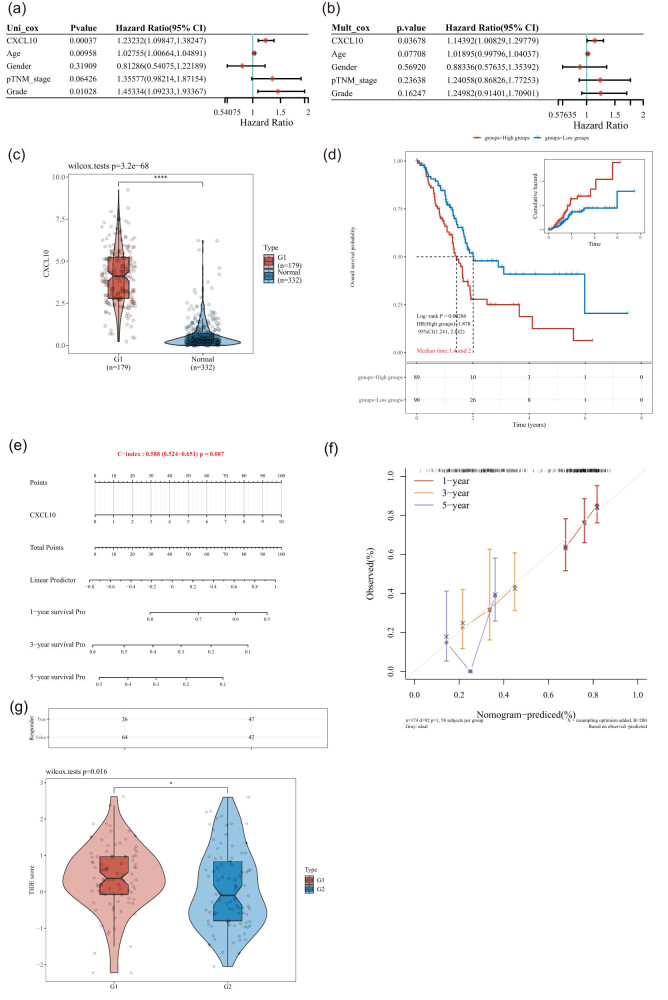
CXCL10 could be a standalone predictor for pancreatic cancer prognosis. (a) and (b) The *p*-value, HR, and CI are analyzed by univariate and multivariate Cox regression. (c) Expression of CXCL10 in tumor tissue (G1) and adjacent tissue (Normal) of pancreatic cancer in TCGA and GTEx databases, different colors represent different groups, and the top-left represents the significant *p*-value. (d) Kaplan–Meier survival analysis of the CXCL10 from TCGA dataset, comparison among different groups was made by log-rank test. HR represents the HR of the low-expression sample relative to the high-expression sample. HR > 1 indicates that the gene is a risk factor, and HR < 1 indicates that the gene is a protective factor. HR (95% Cl) and the median survival time (LT50) for different groups. (e)–(g) Nomograms can predict the 1-, 2- and 3-year overall survival of pancreatic cancer patients. The calibration curve for the overall survival nomogram model in the discovery group. The dashed diagonal line represents the ideal nomogram, and the blue, red, and orange lines represent the 1-, 2- and 3-year of the observed nomogram. The distribution of immune response scores in different groups in the prediction results, different colors represent expression trends in different samples. *p  <  0.05, **p  <  0.01, ***p  <  0.001, and ****p  <  0.0001, asterisks represent the level of significance (*p). The significance of two groups of samples is tested using the Wilcoxon test.

CXCL10, also known as C-X-C chemokine 10 or interferon-induced protein 10, is crucial in mediating immune responses, particularly in inflammation [28,29]. Univariate and multivariate Cox analyses revealed that CXCL10 may be an independent prognostic factor for pancreatic cancer compared to variables such as the patient’s gender, age, and tumor node metastasis stage and grade (Figure 5a and b). To further explore the correlation between CXCL10 and pancreatic cancer risk, TCGA and Genotype-Tissue Expression (GTEx) databases were used to analyze the expression of CXCL10 in pancreatic cancer patients and healthy people (Figure 5c). The results found that the expression of CXCL10 in pancreatic cancer patients was significantly high, higher expression of CXCL10 was linked to worse prognosis in pancreatic cancer patients (Figure 5d). The nomogram model, as evidenced by the calibration curve, demonstrated the strong predictive performance of CXCL10 (Figure 5e and f). The TIDE algorithm effectively forecasts the response of individual samples or subtypes to immune checkpoint inhibitors (Figure 5g). Our findings suggested that patients with elevated CXCL10 expression levels exhibited higher TIDE scores, indicating a diminished response to immune checkpoint inhibitors in this population.

### 3.6 CXCL10 is non-casually linked to the risk of pancreatic cancer

**Figure 6:**
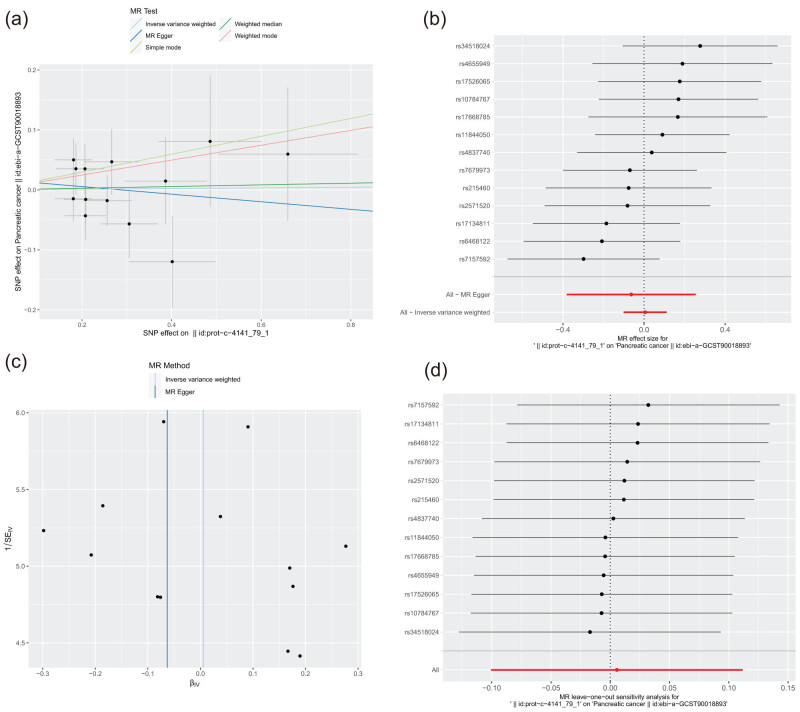
CXCL10 is non-casually linked to the risk of pancreatic cancer. (a) Scatter plot illustrating the causal effect of CXCL10 on the risk of pancreatic cancer. (b) Forest plot depicting the causal effect of each SNP on the risk of trigeminal neuralgia. (c) Funnel plots to visualize the overall heterogeneity of MR estimates for the effect of CXCL10 on pancreatic cancer. (d) Leave-one-out plot to visualize the causal effect of CXCL10 on pancreatic cancer risk when leaving one SNP out.

Previous studies have not demonstrated a causal relationship between CXCL10 and pancreatic cancer progression. Therefore, we used MR to explore the correlation. Table S4 details the SNP characteristics of CXCL10 concerning pancreatic cancer, establishing that no SNPs served as weak instrumental variables. The potential causal impact of each SNP on pancreatic cancer risk is depicted in Figure 6a and b. Various statistical methods, including inverse-variance weighted, MR–Egger, weighted median, and simple and weighted mode, indicated that there might not be a direct causal relationship between CXCL10 expression and pancreatic cancer (Figure 6c). Comprehensive MR analysis was conducted on the residual SNPs after excluding each one sequentially (Figure 6d), and the outcomes remained stable; all SNPs’ influence was significant for causality. This consistency implied the absence of any single dominant SNP influencing the relationship between CXCL10 levels and pancreatic cancer, affirming the reliability of the initial MR findings.

### 3.7 CXCL10 enhances pancreatic cancer metastasis by promoting M2 polarization in macrophages

**Figure 7:**
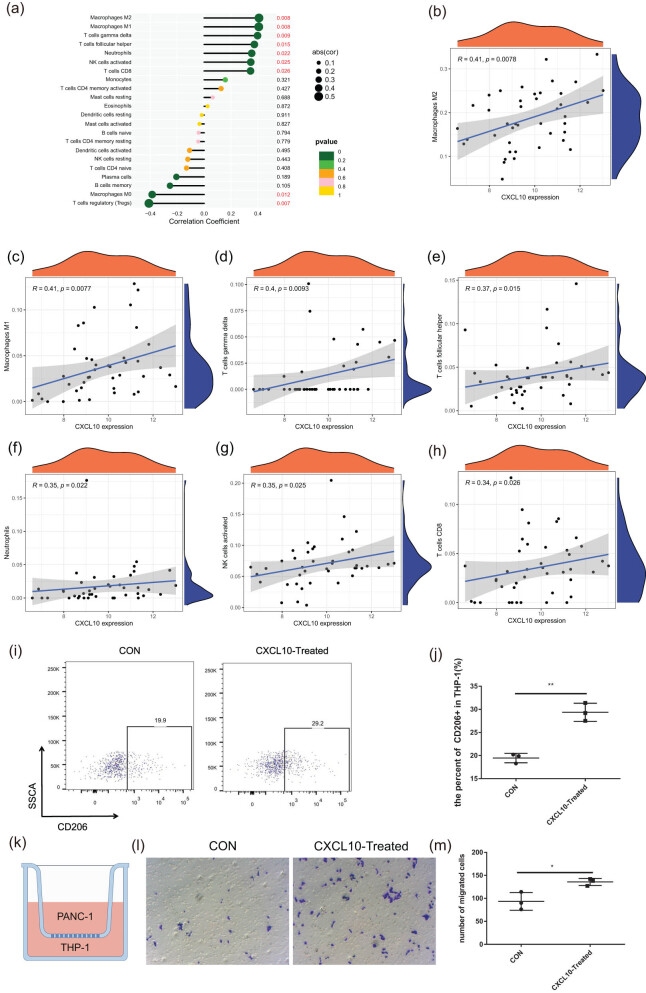
CXCL10 can enhance pancreatic cancer metastasis by promoting M2 polarization in macrophages. (a) Correlation analysis between CXCL10 and immune cells. (b)–(h) Correlation analysis between CXCL10 and immune cells. (i–j) Proportion of M1 and M2 macrophages analyzed by flow cytometry assay. (k) Schematic diagram of cell co-culture. (l) and (m) Representative image of Transwell co-culture of PANC-1 and THP-1 cells.

CXCL10, as an essential chemokine, directly or indirectly affects tumor growth and metastasis by regulating the differentiation and migration of immune cells [28]. Consequently, we investigated whether CXCL10 affects the progression of pancreatic cancer by regulating immune cells. In pancreatic cancer, high levels of CXCL10 were associated with elevated levels of M2 and M1 macrophages, gamma delta T cells, follicular helper T cells, neutrophils, activated natural killer cells, and CD8^+^ T cells when compared to low CXCL10 expression groups (Figure 7a–h). Further analysis revealed a positive correlation between CXCL10 and the proportion of M2 macrophages (Figure 7b). Flow cytometry found that CXCL10 could stimulate macrophage polarization toward the M2 type (Figure 7i and j). As shown in Figure 7k, in a co-culture experiment utilizing CXCL10-treated THP-1 and PANC-1 cells, an increase in PANC-1 cell metastasis was observed (Figure 7l and m). These findings highlight a significant link between CXCL10 and the infiltration of various immune cells in pancreatic cancer.

### 3.8 CXCL10 may promote macrophage polarization toward M2 by regulating VEGFA expression

**Figure 8:**
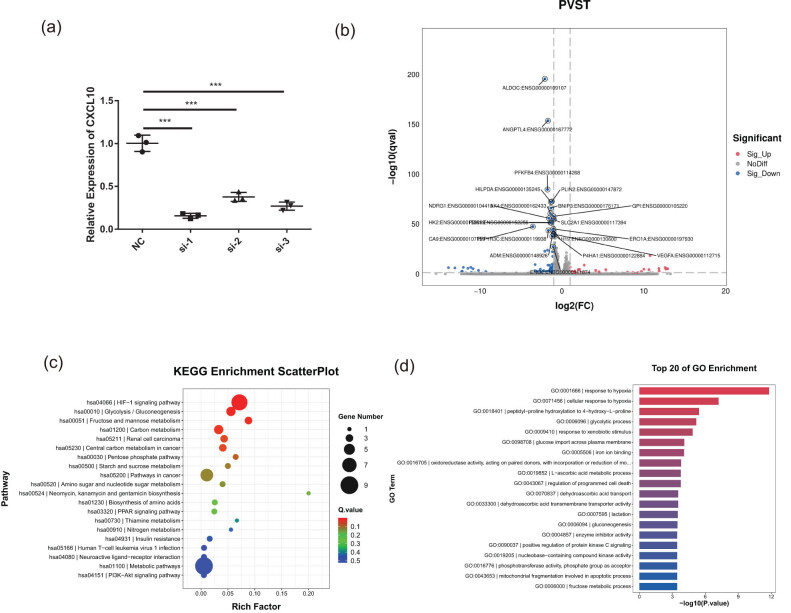
CXCL10 may promote macrophage polarization toward M2 by regulating VEGFA expression. (a) CXCL10 was effectively silenced in PANC-1 cells transfected with si-1 by qRT-PCR compared to si-control. (b) Gene expression of PANC-1 cells after CXCL10 knockdown: red indicates genes with increased expression in the NC group and blue indicates genes with decreased expression. (c) The KEGG analysis of DEGs between NC and si-1 (PANC-1). (d) The GO analysis of DEGs between NC and si-1 (PANC-1).

We employed siRNA to knock down CXCL10 expression in PANC-1 cells to elucidate the CXCL10 role in macrophage polarization toward the M2 phenotype. Moreover, the si-1 knockdown demonstrated the highest efficacy (Figure 8a). Thus, the si-1 group was selected for subsequent analyses. Transcriptome sequencing was conducted on cells from the NC and the si-1 groups. VEGFA expression was significantly reduced in the si-1 group compared to the NC group (Figure 8b). VEGFA facilitates macrophage polarization toward the M2 phenotype within the tumor microenvironment [29,30]. KEGG enrichment analysis (Figure 8c) indicated significant enrichment of signaling pathways such as hypoxia-inducible factor-1 alpha (HIF-1), PI3K-Akt, and PPAR, which are known to promote macrophage M2 polarization [30–32] within the NC group. GO enrichment analysis revealed the predominant association between differential genes and hypoxia response signaling pathways in the NC group (Figure 8d). These findings suggested that CXCL10 may facilitate M2 polarization of macrophages via regulating VEGFA and potentially modulating macrophage activity by influencing pathways, such as HIF-1 and PI3K-Akt.

## 4 Discussion

Pancreatic cancer is a severe illness and is challenging to identify and manage [33]. Pancreatic cancer remains a major global health challenge due to its typically aggressive nature and poor prognosis. Early detection and innovative treatment strategies are crucial in improving the survival rates of the patients. This study aimed to unravel the molecular underpinnings of pancreatic cancer, employing a multi-faceted approach that included DEG analysis, network construction, and causal inference analyses. This study found intricate regulatory associations between genes and potential targets for better treating pancreatic cancer.

In our research, we first used WGCNA analysis to screen the hub genes of pancreatic cancer. We identified eight genes, including SPEN, PITX1, KIF6, MSX2, DLX1, CXCR3, TRIM56, and CXCL10 as the candidate hub genes for pancreatic cancer. Our nomogram model displayed outstanding performance in predicting pancreatic cancer. Subsequently, we calculated ROC curves for five hub genes (MSX2, PITX1, CXCL10, SPEN, and KIF6) to evaluate the diagnostic effect. The AUC of the nomogram can differentiate pancreatic cancer from the control group. These results were consistent with previous observational studies, Ma et al. [34] found that SPEN gene mutation occurred in pancreatic cancer patients and is related to the progress of pancreatic cancer, MSX2 [35] and DLX1 are vital in advancing pancreatic cancer [36].

Univariate and multivariate Cox analyses demonstrated that CXCL10 may serve as an independent prognostic factor for pancreatic cancer. Consistent with previous studies [37,38], the expression of CXCL10 was increased in pancreatic cancer and linked with poor prognosis. CXCL10 is a small molecule chemokine that primarily functions through its receptor CXCR3 [39]. The role of CXCL10 and its receptor CXCR3 in tumorigenesis and tumor progression was complex and multifaceted [40]. Accordingly, CXCL10 may be used as an independent prognostic indicator for pancreatic cancer. Previous studies investigated the correlation between CXCL10 and pancreatic cancer; however, causal analysis and specific mechanism exploration remained unexplored. This study used MR analysis to find the potential causal link between CXCL10 and pancreatic cancer. Insufficient evidence was found using current genetic tools and statistical models to prove that the variations of CXCL10 directly influence disease occurrence. However, these findings should not be interpreted as indicating that genetic factors lack influence on disease risk. Instead, it could be due to insufficient sample sizes, suboptimal selection of genetic tools, or the complex and variable genetic determinants of the disease. Future research may need to consider more genetic variations, larger sample sizes, or different statistical methods to further explore this issue.

Nonetheless, the role and underlying mechanisms of CXCL10 in the progression of pancreatic cancer remain inadequately understood. Zohar found that CXCL10 could acidify and polarize forkhead box protein 3 negative type 1 regulatory T cells (Treg) or T helper cells17 (Th17) from naive T cells through STAT1, STAT4, and STAT5 [41]. CXCL10 stimulates immune cells by polarizing and activating T helper cell 1, allowing them to enter tumor tissue and exert phagocytic effects [42]. We observed that CXCL10 was positively correlated with the proportion of M2-type macrophages. Flow cytometry and co-culture experiments demonstrated that CXCL10 could promote the migration of pancreatic cancer cells by inducing M2 polarization of macrophages. Chemokines serve as the primary mediators of macrophage chemotaxis [43,44]; however, the mechanisms underlying their regulation of macrophage polarization into M1 and M2 phenotypes remain unclear. Previous studies have indicated that CCL19, CCL21, CCL24, CCL25, CXCL8, and CXCL2 specifically induce macrophage polarization towards the M1 phenotype, while CCL7 facilitates polarization towards both M1 and M2 phenotypes [45]; nevertheless, the mechanism by which CXCL10 induces the polarization of macrophages towards the M2 phenotype in the context of pancreatic cancer has not yet been elucidated. Previous research has demonstrated that the activation of signaling pathways, including JAK1/STAT3, PPAR, and AMPK/mTOR, can facilitate the polarization of macrophages toward the M2 phenotype [46–49]. Furthermore, lactate produced by tumor cells has been shown to enhance the expression of genes such as VEGFA and Arg1 via HIF-1α, thereby promoting M2 macrophage polarization [30]. The transcriptome sequencing results found that CXCL10 could regulate the expression of VEGFA; moreover, compared to the CXCL10 knockout group, signaling pathways such as PPAR and HIF-1 were significantly enriched in the CXCL10 high expression group. As a result, it was speculated that CXCL10 affects macrophage polarization by regulating the expression of VEGFA. However, the limitation is that we were unable to verify this further. Consequently, we hypothesize that CXCL10 may facilitate the progression of pancreatic cancer by inducing macrophage polarization towards the M2 phenotype through the upregulation of VEGFA. However, the precise mechanisms by which CXCL10 regulates VEGFA expression in pancreatic cancer remain unclear, necessitating further investigation in subsequent studies.

Notably, certain studies indicate that, under specific tumor types and microenvironmental conditions, CXCL10 may play a role in inhibiting tumor progression by facilitating immune cell chemotaxis, suppressing angiogenesis, and promoting apoptosis in tumor [50,51]. The role of CXCL10 in tumor progression is a double-edged sword, and its specific effects depend on various factors, including tumor type, characteristics of tumor microenvironment, and immune system status. Additional investigation is required to elucidate the impact of CXCL10 on advancing or impeding the development of particular tumor varieties.

## 5 Conclusion

Using a co-expression network based on WGCNA, we discovered hub genes associated with pancreatic cancer. CXCL10 demonstrates significant potential as a therapeutic target in cancer treatment, warranting further investigation in the future diagnosis and management of pancreatic cancer.

## Abbreviations

CXCL10: chemokine (C-X-C motif) ligand 10
DEGs: differentially expressed genes
GEO: gene expression omnibus
GO: gene ontology
GWAS: genome-wide association study
ICB: immune checkpoint blockade therapy
IP-10: interferon-inducible protein 10
KEGG: Kyoto Encyclopedia of Genes and Genomes
MR: Mendelian randomization
PPI: protein–protein interaction
ROC: receiver operating characteristic curve
SNPs: single nucleotide polymorphisms
WGCNA: weighted gene co-expression network analysis

## Supplementary Material

Supplementary Table
